# Relationship of Asymmetrical Dimethylarginine, Nitric Oxide, and Sustained Attention during Attack in Patients with Major Depressive Disorder

**DOI:** 10.1155/2014/624395

**Published:** 2014-01-16

**Authors:** Serpil Canpolat, İsmet Kırpınar, Erdem Deveci, Hülya Aksoy, Zafer Bayraktutan, İbrahim Eren, Recep Demir, Salih Selek, Nazan Aydın

**Affiliations:** ^1^Department of Psychiatry, Çumra State Hospital, 42500 Konya, Turkey; ^2^Department of Psychiatry, School of Medicine, Bezmialem Vakif University, 34093 Istanbul, Turkey; ^3^Department of Biochemistry, School of Medicine, Atatürk University, 25240 Erzurum, Turkey; ^4^Department of Psychiatry, Konya Training and Research Hospital, 42090 Konya, Turkey; ^5^Department of Neurology, School of Medicine, Atatürk University, 25240 Erzurum, Turkey; ^6^Department of Psychiatry, School of Medicine, Medeniyet University, 34722 Istanbul, Turkey; ^7^Department of Psychiatry, School of Medicine, Atatürk University, 25240 Erzurum, Turkey

## Abstract

We investigated the relationship of serum nitric oxide (NO) and asymmetrical dimethylarginine (ADMA) levels with cognitive functioning in patients with major depressive disorder (MDD). 41 MDD patients (Beck depression scale scores >16) and 44 controls were included in the study. Rey verbal learning and memory test, auditory consonant trigram test, digit span test, Wisconsin card sorting test, continuous performance task (TOVA), and Stroop test scores were found to be impaired in patients with major depressive disorder when compared to healthy controls. There was no significant difference between patient and control groups in terms of serum NO and ADMA. Serum NO levels were correlated with TOVA test error scores and Stroop test time scores, whereas serum ADMA levels were negatively correlated with TOVA test error scores. Metabolic detriments especially in relation to NO metabolism in frontal cortex and hypothalamus, psychomotor retardation, or loss of motivation may explain these deficits.

## 1. Introduction

Major depressive disorder (MDD) is a heterogenous disorder showing psychological, behavioral, and physiologic signs and symptoms [1]. In MDD, cognitive functions are also altered in addition to the symptoms such as mood and behavioral changes. Deteriorations were reported in executive functions, visual-motor attention, short-term memory, working memory, verbal and nonverbal learning, and verbal fluency [2–5]. Although some studies reported memory impairment as the main problem in MDD [6–8], some others reported executive function impairment as the main problem [3].

Nitric oxide (NO) is a colourless, toxic gas with a short half-life. It is lipophilic and quite stable in lack of oxygen [9, 10]. NO is a widely used signal molecule in the nervous system [11]. Its molecular weight is very low and it may affect the control of dopamine release. NO is found to be related to learning and memory, anxiety, schizophrenia, mood disorders, obsessive compulsive disorder, and eating disorders [12–16]. NO has some roles in emotional, behavioral, and cognitive processes. Some studies reported that NO levels were elevated in patients with MDD and turned back to normal levels after treatment [17, 18]. On the contrary, some other studies reported reduced plasma NO levels which were increased after treatment [19, 20]. Animal studies showed the effects of nitric oxide synthase (NOS) inhibitors on learning and memory [21, 22]. NOS activity was found to be related to spatial memory [23].

NOS induces NO synthesis from L-arginine. Asymmetrical dimethyl arginine (ADMA) is the endogenous competitive inhibitor of NOS [24]. Some lines of evidence suggest a relationship between ADMA levels and cognitive impairment in Alzheimer's disease [25]. ADMA is metabolized by the enzyme dimethyl arginine dimethylaminohydrolase (DDAH). It has been reported that protecting DDAH activity in neurons and reducing ADMA accumulation were important in Alzheimer's disease and cognitive impairment due to aging [20]. Increase in ADMA levels was associated with impairment of spatial memory in rats [26]. In this study, we aimed to investigate the relationship of cognitive functions and serum levels of NO and ADMA in patients with MDD.

## 2. Materials and Methods

### 2.1. Study Groups

Consecutive patients who were diagnosed as MDD based on DSM-IV criteria having at least 17 points in Beck depression scale and gave written informed consent were included in the study. Control group was formed by hospital staff, students, and relatives of patients. Inclusion criteria were major depressive patients during depressive episode aged between 18 and 65 years. Exclusion criteria were severe organ failure, drug dependence (apart from smokers), antioxidant use, infection, severe obesity, psychotropic drug use in the last two weeks, epilepsy, severe neurologic deficit, cardiac disorders, diabetes mellitus, hypercholesterolemia, any DSM-IV diagnosis apart from depressive disorder, treatment with anticonvulsants, antiaggregants, antihypertensives, antioxidants, antidiabetics, nonsteroid anti-inflammatory drugs and antihyperlipidemics, dementia, and mental retardation. Patients and controls who did not give written informed consent were also excluded.

### 2.2. Procedure and Instruments

#### 2.2.1. Sociodemographic Data Form

This form was developed by the researcher. It covers information such as age, gender, education level, health history, ongoing or previous psychiatric disorders and drug history.

#### 2.2.2. Beck Depression Inventory (BDI)

In this study, we used 1978 version of the BDI adapted by Hisli [27].

#### 2.2.3. Structured Clinical Interview for DSM Disorders (SCID)

Reliability of the Turkish version was studied by Çorapçıoğlu et al. [28] in 1999.

### 2.3. Neurocognitive Assessment

The neurocognitive battery included the Rey auditory verbal learning test (RAVLT), auditory consonant trigram test (ACTT), controlled oral word association test (COWAT), digit span test (DST), trail making test A/B (TMT-A/B), Wisconsin card sorting test (WCST), Stroop test (ST), and test of variables of attention (TOVA). All these measurements were performed in the laboratory of in-patient treatment clinic. Optimal silence, illumination, and isolation were obtained. All tests were performed by psychiatrists. All tests of the same participant were completed on the same day. WCST and TOVA tests were performed by computer software.

#### 2.3.1. RAVLT

This test evaluates verbal episodic memory and learning. A list of 15 words is read and after the first 5 readings, the patient is asked to repeat these words. Total learning score is total word count remembered between 1 and 5 trials. At the 6th and 7th trials, the patient is asked to repeat the words without reading the list (late memory score). In the recognition session, the patient is asked to recognize those 15 words in a larger list (correct recognition score). The correct distinguishing score is calculated by the formula (true positive + true negative/50) [29, 30]. The score of 1 depicts excellent result whereas 0 depicts the worst result.

#### 2.3.2. ACTT

This test is used to assess working memory. After giving three consonants, the patient is asked to count down from a certain number. Then, in certain intervals, the patient is stopped and asked to remember the consonants. The total number of remembered letters is recorded [31].

#### 2.3.3. COWAT

This test is used to assess verbal fluency and the patient is asked to produce words starting with the letters K, A, and S in 60 seconds. These letters were used in Turkish standardization study [32].

#### 2.3.4. DST

This test is used to assess verbal attention and digital processing memory (working memory). This test comprises “forward” and “backward” sections that digits should be told from start-to-end or from end-to-start, respectively. Simply, the patient is asked to repeat each digit in single steps with increasing 1 order. The “forward” part of the test assesses verbal attention whereas the “backward” part evaluates working memory [31].

#### 2.3.5. TMT-A/B

This test is used to assess frontal executive functions, attention, mental flexibility, visual pursuit, and motor speed [31]. In the A section, the patient is asked to count digits between 1 and 25, whereas, in B section, the patient is asked to count digits and letters such as 1-A–2-B [33]. Total time is recorded.

#### 2.3.6. ST

This test is used to assess frontal executive functions and attention. The patient is asked to read colour names written in black and then written colour names first followed by colour of ink. Total reading time is recorded [31].

#### 2.3.7. WCST

This test assesses executive functions, such as cognitive flexibility as well as problem solving and abstraction abilities. Participants had to classify a series of cards into 3 categories in 128 cards, after having found the classification rule (color, number, or forms) [34, 35]. We used computerized form of the test (WCST: CV4).

#### 2.3.8. TOVA

This test is a computerized continuous performance test assessing sustained attention. The subjects were asked to push a button connected to a computer when they recognized the target on the monitor for an uninterrupted period of 20 min. The target means a small square appearing on the upper part of a rectangle. The nontarget means a small square appearing on the bottom of the rectangle. A stimulus flashed on a screen every 2 seconds. The target is presented on 22.5% and 77.5% of the trials during the first and second halves, respectively. The data was obtained in the domains of the omission error (inattention), the commission error (impulsivity), the response time, and the variability. All the variables are recorded for each 5 min quarter and 10 min halves, as well as the overall total scores for each variable. The scores are compared to the standardized norms, and the interpretation of data is reported in a printable form [30, 36].

### 2.4. Biochemical Measurements

Blood samples were collected after a 12-hour fasting period in the morning between 9.00 and 12.00 hours. After centrifugation, serum samples were allocated into Eppendorf tubes and stored at −80 C° until assay.

#### 2.4.1. NO Measurement

NO measurement was performed on nitrite levels as NO reacts with oxygen and converts into nitrite and nitrate. In this method, nitrate molecules were converted into nitrite by nitrate reductase. Then, nitrite measurement was done by Griess reaction [37].

#### 2.4.2. ADMA Measurement

We used high performance liquid chromatography (HPLC) to measure serum ADMA concentration. Samples were measured by derivating pre-column OPA (o-pthalaldehyde) [38].

### 2.5. Statistical Analysis

The values were presented as mean ± standard deviation (SD) or percentages where appropriate. The normality distribution of variates was tested by Kolmogorov-Smirnov and Shapiro-Wilk tests. Intergroup comparisons were made by Student's *t*-test. To test correlations, bivariate Pearson correlation analysis was used. All statistics were reported in two-tailed form. A *P* value lower than 0.05 was accepted as statistically significant.

## 3. Results

In total, 104 participants were enrolled. Forty-one patients with major depression and 44 healthy controls completed the study. Drop-out reasons were long duration of tests in 8 participants, problems in blood sampling in 9 subjects, and violation of study protocol (drug use) in 2 subjects. Ten subjects quitted without any reason.

Sociodemographic data and smoking status of patient and control groups were given in Table 1. The patient and control groups were similar in terms of smoking status and sociodemographic characteristics. The mean number of attacks of patients was 1.41 ± 0.71 and the mean score of Beck depression scale was 30.49 ± 0.38. Patients with MDD showed significantly worse results in all tests of the test battery when compared to controls (Table 2). The mean serum NO and ADMA levels were similar in both MDD and control groups (Table 3).

Serum NO levels were inversely correlated with success rates in ST, WCST, and TOVA. Serum ADMA levels were inversely correlated with TOVA test commission and omission error scores. In other words, the higher the serum ADMA levels, the higher the success rate in TOVA test (Table 4).

## 4. Discussion

NO shows a free radical structure and is related to learning and memory, feeding behavior, and mood disorders. Herken et al. [18] failed to find any significant difference between MDD patients and healthy controls in terms of nitrate levels. Some previous studies reported NO increase in MDD [17], whereas others found lower plasma NO levels in MDD patients [19, 20]. In our study, NO levels were similar in MDD and healthy controls. NO levels were also studied in a group of patients with suicide attempt. Plasma NO levels were significantly higher in MDD patients who committed to suicide than in MDD patients without suicide and in healthy controls [39]. Previous studies reported an association between serum NO levels and eating behavior [16], anxiety [13], and suicide attempt [39].

Animal studies showed the effects of NOS inhibitors on learning and memory [21]. In our study, NO levels were not in relation to memory, learning, attention speed, divided attention, verbal attention, and verbal fluency. But TOVA test scores were correlated with serum NO levels. In addition, there was a linear correlation between serum NO levels and time scores of ST. This test is accepted as the “gold standard” for assessing ability to focus and maintain attention. The literature search showed no previous study on the relationship of serum NO levels with sustained attention performance and ability to focus.

The enzyme NOS was shown to be in cerebral cortex [13]. Prefrontal region plays a role in sustained attention [40]. NO is a free radical and shows an important neurotoxicity. Zhang et al. [41] showed that NOS increase in hippocampal cell lines induced NO elevation which in turn stimulated expression of proapoptotic Bax gene and mitochondrial-mediated apoptosis. Taken together with our results, we suggest that NO increase may negatively affect the functioning of the neurons of prefrontal cortex and may impair ability to continue attention.

The mean serum ADMA levels, an endogenous competitive inhibitor of endothelial NOS, were similar in both MDD and control groups. Conversely, Selley [20] found that plasma NO levels were higher and ADMA levels were lower in 25 MDD patients when compared to 25 healthy controls. He also found a negative correlation between plasma NO and ADMA levels. We failed to find such a correlation. There was no relation between serum ADMA levels and clinical variates in our study. Huang et al. [26] suggested a correlation between the increase in ADMA levels and the impairment of spatial memory in rats. Arlt et al. [25] found a decrease in ADMA levels in cerebrospinal fluid (CSF) of Alzheimer's patients and they suggested a relationship between CSF ADMA levels and deterioration of cognitive functions. In another study, Abe et al. [42] evaluated serum ADMA levels and mini-mental test in Alzheimer's patients and they reported a correlation between serum ADMA decrease and cognitive functions. In contrast, we failed to find any relationship between ADMA levels and cognitive functions such as memory, learning, attention, verbal attention, verbal fluency, and executive functions.

There are several limitations of this study. First, our neurocognitive battery did not include cognitive tests assesing social cognition, visual span memory and visual attention. Adding these parameters would increase the validity of our results. But our battery including SCID, Beck depression scale, and other measurements could be completed in 1.5 to 2 hours. In order to complete all the scales and questionnaires on the same day, we choose not to add above-mentioned tests. Tests that need longer time to complete may also interact with cognitive functions and lead to inaccurate results. No previous study evaluated the relationship of NO and ADMA levels with neuropsychiatric test scores in patients with MDD. We used a wide range of these tests.

## 5. Conclusions

In conclusion, we suggest that NO increase and ADMA decrease are related to deficits in sustained attention in MDD patients. To our knowledge, this is the first study which investigated both ADMA and NO levels in relation to cognitive tests in major depressive disorder.

## Figures and Tables

**Table 1:** Comparison of study group with healthy controls in terms of age, gender, years of education, and smoking status.

	MDD group (*n* = 41)	Control group (*n* = 44)	*P*	df	*t*	*χ* ^2^
Age (years)	26.27 ± 4.87	27.07 ± 4.30	0.42	83	−0.80	
Gender (%)			0.99	1		0
Female	28 (68.3%)	30 (68.2%)				
Male	13 (31.7%)	14 (31.8%)				
Education (years)	12.85 ± 3.28	12.70 ± 4.59	0.86	77.90	0.17	
Smoking status			0.84			
Nonsmokers	26 (63.4%)	27 (61.4%)		1		0.38
Smokers	15 (36.6%)	17 (38.6%)				

MDD: major depressive disorder.

**Table 2:** Comparison of study group with healthy controls according to cognitive tests.

Neurocognitive tests	MDD group (*n* = 41)	Control group (*n* = 44)	*P*	*t*	df
Rey verbal learning and memory test					
Total learning scores (1–5)**	46.44 ± 6.59	55.66 ± 6.09	0.0001**	−6.70	83
Delayed recalling scores (7)**	9.54 ± 1.98	12.02 ± 1.78	0.0001**	−6.07	83
True positives**	13.39 ± 1.77	14.29 ± 0.79	0.004**	−3.00	54.59
Recognition percent correct score	0.90 ± 0.13	0.94 ± 0.12	0.168	−1.39	83
Auditory consonant trigram test total scores**	44.51 ± 6.80	53.77 ± 3.92	0.0001**	−7.61	63.04
Controlled word association test total scores**	29.31 ± 12.12	42.52 ± 12.72	0.0001**	−4.89	83
Digit span test (DST)					
Forward section score**	5.82 ± 1.99	7.81 ± 1.74	0.0001**	−4.89	83
Backward section score**	5.46 ± 2.32	8.11 ± 2.54	0.0001**	−5.00	83
Total scores*	12.85 ± 7.78	15.93 ± 4.00	0.023*	−2.31	83
Trail making test (TMT) (seconds)					
Part A**	51.85 ± 13.41	37.49 ± 12.19	0.0001**	5.17	83
Part B**	143.53 ± 121.53	72.87 ± 33.13	0.001**	3.60	45.52
Stroop test main card reading time (seconds)**	31.83 ± 9.01	19.79 ± 4.54	0.0001**	7.68	58.18
Wisconsin card sorting test (WCST)					
Categories completed*	3.98 ± 2.26	5.05 ± 1.53	0.014*	−2.53	69.84
Total correct percentage score**	62.26 ± 16.19	72.22 ± 17.73	0.008**	−2.69	83
Perseverative errors**	20.41 ± 11.90	13.72 ± 8.96	0.004**	2.93	83
Failure to maintain set**	1.24 ± 1.39	0.55 ± 0.69	0.005**	2.89	57.93
TOVA test scores					
Omission errors**	10.73 ± 19.74	1.41 ± 2.03	0.004**	3.00	40.79
Commission errors	13.02 ± 13.30	10.89 ± 14.97	0.49	0.69	83

*P < 0.05; **P < 0.01; MDD: major depressive disorder.

**Table 3:** Comparison of study group with healthy controls according to blood NO and ADMA levels.

	MDD group (*n* = 41)	Control group (*n* = 44)	*P*	*t*	df
NO levels (*µ*mol/L)	35.64 ± 28.52	28.38 ± 25.35	0.21	1.24	83
ADMA levels (*µ*mol/L)	7.33 ± 5.29	6.80 ± 4.05	0.60	0.52	83

NO: nitric oxide; ADMA: asymmetrical dimethyl arginine; MDD: major depressive disorder.

**Table 4:** Correlations between cognitive test scores and blood NO and ADMA levels.

Neurocognitive tests	NO		ADMA	
	*r*	*P*	*r*	*P*
Rey verbal learning and memory test				
Total learning scores (1–5)	−0,08	0,44	−0,03	0,77
Delayed recalling scores (7)	−0,09	0,40	−0,05	0,62
True positives	−0,06	0,59	0,06	0,57
Recognition percent correct score	−0,02	0,84	0,09	0,40
Auditory consonant trigram test total scores	−0,11	0,33	−0,02	0,85
Controlled word association test total scores	−0,08	0,46	−0,13	0,22
Digit span test (DST)				
Forward section score	−0,20	0,07	0,20	0,06
Backward section score	−0,17	0,12	0,07	0,51
Total scores	−0,08	0,46	0,18	0,13
Trail making test (TMT)				
Part A	0,16	0,14	0,02	0,83
Part B	0,03	0,78	0,09	0,39
Stroop test main card reading time	0,21	0,04*	0,02	0,81
Wisconsin card sorting test (WCST)				
Categories completed	−0,02	0,81	−0,27	0,80
Total correct percentage score	−0,61	0,57	0,02	0,82
Perseverative errors	0,02	0,87	−0,08	0,44
Failure to maintain set	0,20	0,04	0,15	0,16
TOVA test scores				
Omission errors	0,01	0,95	−0,12	0,0001*
Commission errors	0,20	0,001*	−0,06	0,04
Response time mean	−0,04	0,20	−0,02	0,60
Commission response time mean	−0,28	0,0001*	0,04	0,24
D Prime	−0,08	0,01	0,06	0,05
Postcommission response time	0,02	0,47	−0,24	0,0001*

*P < significance level set after Bonferroni correction; *r*: correlation coefficient; NO: nitric oxide; ADMA: asymmetrical dimethyl arginine (the data are from major depressive patients group).
